# Supplementary material for: Chromothripsis during telomere crisis is independent of NHEJ, and consistent with a replicative origin
Source: Genome Res. 2019 May;29(5):737–49. doi: 10.1101/gr.240705.118 (PMC6499312; doi:10.1101/gr.240705.118)
Supplement: Supplemental Material [file supp_gr.240705.118_Supplemental_file_1.zip › contigs/annotated_contigs/DB57/contig.3.DB57_length_363_mean_cov_17.6005509642.docx]

**DB57_length_363_mean_cov_17.6005509642**

```
CCACTGGCCTGCGCCCACTGCCTCGCGCTCCCTAGTAAGCGGCACCCAGTACCTCAGGTGGAAATGCAGAAATCACCCGTCTTCTGTGT
 >chr10:18325454-18325702 + E=8e-108
CGCTCACGCTGGGAGCTGTAGACTGGAGCTGTTCCTATTCGGCCATCTTGGCTCCTCCCCCGTGATATCTTTTACAACAAAGGCAGCCT

AATAACTATATCATTGGCATCTTGCAGTTAGCATTTTAAAAATTATATATATATATATATATATATATA|T|TTTTTTTTTTTTTTTTT
                                                                      >chr20:56824786-5
TTTTTTTTTTTTTTTTTTTTTTTTTTTTTTTTTG|T|TTTTTTTTTTTTTTTTTTTGTTTTTTTTTTCTTTTTTTTTTTTTTTTTTTTT
6824839 - E=2e-20 p=0e+00          >chr2:33091781-33091845 + E=5e-18
TTTTTTCTTTT
```
